# Editorial: Cancer evolution: From biological insights to therapeutic opportunities

**DOI:** 10.3389/fgene.2022.984032

**Published:** 2022-08-09

**Authors:** Andrew A. Davis, Lorenzo Gerratana, Marco Mina

**Affiliations:** ^1^ Washington University in St. Louis, St. Louis, MO, United States; ^2^ Department of Medical Oncology-CRO Aviano, National Cancer Institute, IRCCS, Aviano, Italy; ^3^ University of Udine, Udine, Italy; ^4^ HAYA Therapeutics, Lausanne, Switzerland

**Keywords:** cancer evolution, biomarker, cancer dynamics, tumor microenevironment, cancer genomics

At present, cancers are described in both biological and clinical settings with static models that characterize tumors by the phenotypic and genotypic features observed at a given time point. However, cancers are highly dynamic processes that evolve based on specific genomic and epigenomic changes. Such dissonance between the model used to describe tumors and their true nature reverberates all the way from basic biological research to clinical practice. For instance, it is well established that anti-cancer treatments impose selective pressures leading to the emergence of resistant clones (Zheng et al., 2016; O’Leary et al., 2018). This evolutionary process is stochastic and access to better models to analyze and predict mechanisms of resistance is critical.

Historically, the first barrier to the characterization of the evolutionary properties of cancer was the limited amount of available data to accurately reflect tumor heterogeneity, which was traditionally based on a single tissue biopsy (Bertucci et al., 2019; Gerratana et al., 2019; Nguyen et al., 2022). Several factors have changed this paradigm, including the advancement of molecular profiling technologies, the substantial decrease in sequencing costs, and the introduction of multimodal serial sampling in clinical and research settings. Multimodal molecular profiling has pushed research in several directions. On the one hand, profiling of circulating tumor cells (CTC) and cell-free DNA (cfDNA) in peripheral blood samples is paving the way toward non-invasive diagnosis and monitoring of cancer progression (Gerratana et al., 2021). On the other hand, multi-regional sequencing, single cell and spatial transcriptomics, as well as newly emerging epigenomic profiling techniques are leading to a renewed interest in the analysis of genomic, transcriptomic, and epigenetic mechanisms that have previously been understudied (Mina et al., 2017; Mastoraki et al., 2018; Sanchez-Vega et al., 2018; Penson et al., 2019; Gerratana et al., 2020; Nguyen et al., 2022).

The articles in this research topic address important aspects of cancer evolution, encompassing models associated with a diversity of tumor types including renal cell carcinoma, glioblastoma, breast, colorectal, lung, ovarian, pancreatic and prostate cancer. Our *a priori* expectation when we proposed this research topic was to be confronted by a barrage of works focusing mainly on the genetic aspects of cancer evolution. Looking retrospectively at the collection of accepted manuscripts, however, we were surprised to observe some specific yet unexpected topics recurrently emerging.

Multiple articles within this collection focused on the role of non-coding RNA in cancer, including in particular microRNA (miRNA), circular RNA (circRNA) and long non-coding RNA (lncRNA) (Duică et al.; Gao et al.; Qi et al.; Li et al.; Zheng et al.; Li et al.; Chen et al.; Zhao et al.). Duică et al. presented a review of the role of miRNAs in cancer-relevant processes, focusing on gynecological malignancies (Duică et al.). Another study (Ming Li et al.) evaluated exosomal miRNAs as a mechanism of resistance in small cell lung cancer with particular elevation of exosomal miR-92b-3p that was associated with the PTEN/AKT pathway based on preclinical models (Li et al.). Importantly, this study also included a clinical cohort of 50 patients to help validate the authors’ hypothesis that downregulation of this biomarker was associated with better clinical outcomes. Junchen Li et al., instead, focused on the anti-cancer mechanism of andrographolide in patients with luminal-like breast cancer through the inhibition of miR-21-5p (Li et al.). Similarly, Chen et al. investigated the role of circular RNA (circRNAs) in prostate cancer. The authors focused on the regulation, expression, and functional effects of circNOLC1 for *in vitro* and *in vivo* models, proposing circNOLC1 as a potential biomarker and target for prostate cancer treatment.

Multiple studies focused on lncRNAs as well. Gao et al. published a general review on lncRNA mechanisms of action, regulatory functions, and biological relevance in tumor development and progression. In a study by Zhao et al., lncRNAs were compared across primary and recurrent glioblastoma tissue to identify mechanisms that drive poor prognosis, a finding that was validated in preclinical models. The authors identified lncRNA NONHSAT079852.2 as a relevant biomarker in glioblastoma, acting as a sponge of hsa-mir-10401-3p and enhancing HSPA1A expression, and promoting tumor cell proliferation, invasion, and recurrence of glioblastoma (Zhao et al.). Qi et al. evaluated lncRNAs as potential biomarkers to assess heterogeneity in metastasis for colon and rectal cancers. The analysis identified two biomarker lncRNAs, KCNQ1OT1 and SNHG1, associating with cancer initiation and metastatic potential. Interestingly, the authors proposed different mechanisms of actions of these two lncRNAs in colon and rectal cancers (Qi et al.). Finally, Zheng et al. identified a panel of prognosis-associated lncRNAs that were significantly associated with survival in ovarian cancer across multiple independent cohorts, including The Cancer Genome Atlas (TCGA) and Gene Expression Omnibus (GEO). The authors further selected and validated the expression of four lncRNAs *in vitro* on multiple ovarian cancer cell lines (Zheng et al.).

A second theme that was recurrent in our collection is the plasticity of the epithelial-mesenchymal transition (EMT). Within this collection, Zheng et al. published a review on the communication between EMT and cancer stem cells (CSC). While this was once thought to be an unidirectional evolution, more recent studies suggest that this transition is bi-directional, stochastic, and mediated by the tumor microenvironment, ultimately showing how such “hybrid state” or “plasticity” is linked to poor prognosis and resistance. Cui et al. studied the role of ENC1 in accelerating EMT processes in colorectal cancer, whereas the study by Shou et al. reported an inverse relation between tissue inhibitor matrix metalloproteinases 1 (TIMP1) and prognosis in samples from patients with renal cell carcinoma. The study evaluated TIMP1 as a biomarker to enhance metastasis via the EMT signaling pathway. Another study explored EMT-related genes using TCGA and local samples for pancreatic ductal adenocarcinoma (Feng et al.). This study identified a 8-gene signature that impoved prediction compared to clinical variables alone, particularly to assess adjuvant treatment response, such as to immune checkpoint inhibitor therapy. In the future, evaluating therapies that target cells in this “hybrid” EMT state may have specific applications to prevent seeding of metastatic sites.

Finally, it is worth mentioning two reviews on protein condensates and RNA modifications. Lu et al. produced a review of Liquid–liquid Phase Separation (LLPS) and protein/nucleic acid condensates. LLPS are a new paradigm in the study of cellular activities recently coming under more intense research focus. Recent progress has been made to understand the roles of LLPS in cancer. Additionally, Dai et al. published a review on RNA modifications and their role in cancer with a deep discussion on the YTH protein family of m6A readers, summarizing the recent advances in structure and biological function of YTH family proteins, and their roles in human cancer and therapy applications.

This collection of articles highlights promises and challenges of characterizing the dynamic aspects of cancer. They nicely expose the critical need for multi-omics biomarkers to track cancer evolution in both research and clinical settings. Interestingly, we observed an emerging interest towards previously less studied molecular players, such as non-coding RNAs, condensates, and RNA modifications, both in the context of cancer cells and tumor microenvironment. We anticipate that the use of integrated models based on multiple biomarkers (epigenomics, genomics, transcriptomics, proteomics, and radiomics) will be necessary to capture the complexity of cancer evolution, especially in today’s clinical settings dominated by a paradigm shift from monotherapy approaches towards combinations of chemotherapy, immune checkpoint inhibitors and targeted therapies.

## References

[B1] Bertucci F. Ng C. K. Y. Patsouris A. Droin N. Piscuoglio S. Carbuccia N. (2019). Genomic characterization of metastatic breast cancers. Nature 569, 560–564. 10.1038/s41586-019-1056-z 31118521

[B2] Gerratana L. Basile D. Franzoni A. Allegri L. Viotto D. Corvaja C. (2020). Plasma-based longitudinal evaluation of ESR1 epigenetic status in hormone receptor-positive HER2-negative metastatic breast cancer. Front. Oncol. 10, 550185. 10.3389/fonc.2020.550185 33072577 PMC7531252

[B3] Gerratana L. Davis A. A. Shah A. N. Lin C. Corvaja C. Cristofanilli M. (2019). Emerging role of genomics and cell-free DNA in breast cancer. Curr. Treat. Options Oncol. 20, 68. 10.1007/s11864-019-0667-9 31256282

[B4] Gerratana L. Davis A. A. Zhang Q. Basile D. Rossi G. Strickland K. (2021). Longitudinal dynamics of circulating tumor cells and circulating tumor DNA for treatment monitoring in metastatic breast cancer. JCO Precis. Oncol. 5, 943–952. 10.1200/PO.20.00345 34136741 PMC8202557

[B5] Mastoraki S. Strati A. Tzanikou E. Chimonidou M. Politaki E. Voutsina A. (2018). ESR1 methylation: A liquid biopsy–based epigenetic assay for the follow-up of patients with metastatic breast cancer receiving endocrine treatment. Clin. Cancer Res. 24, 1500–1510. 10.1158/1078-0432.CCR-17-1181 29284708

[B6] Mina M. Raynaud F. Tavernari D. Battistello E. Sungalee S. Saghafinia S. (2017). Conditional selection of genomic alterations dictates cancer evolution and oncogenic dependencies. Cancer Cell 32, 155–168. e6. 10.1016/j.ccell.2017.06.010 28756993

[B7] Nguyen B. Fong C. Luthra A. Smith S. A. DiNatale R. G. Nandakumar S. (2022). Genomic characterization of metastatic patterns from prospective clinical sequencing of 25, 000 patients. Cell 185, 563–575. e11. 10.1016/j.cell.2022.01.003 35120664 PMC9147702

[B8] O’Leary B. Cutts R. J. Liu Y. Hrebien S. Huang X. Fenwick K. (2018). The genetic landscape and clonal evolution of breast cancer resistance to palbociclib plus fulvestrant in the PALOMA-3 trial. Cancer Discov. 8, 1390–1403. 10.1158/2159-8290.CD-18-0264 30206110 PMC6368247

[B9] Penson A. Camacho N. Zheng Y. Varghese A. M. Al-Ahmadie H. Razavi P. (2019). Development of genome-derived tumor type prediction to inform clinical cancer care. JAMA Oncol. 6, 84–91. 10.1001/jamaoncol.2019.3985 PMC6865333 31725847

[B10] Sanchez-Vega F. Mina M. Armenia J. Chatila W. K. Luna A. La K. C. (2018). Oncogenic signaling pathways in the cancer Genome Atlas. Cell 173, 321–337. e10. 10.1016/j.cell.2018.03.035 29625050 PMC6070353

[B11] Zheng D. Ye X. Zhang M. Z. Sun Y. Wang J. Y. Ni J. (2016). Plasma EGFR T790M ctDNA status is associated with clinical outcome in advanced NSCLC patients with acquired EGFR-TKI resistance. Sci. Rep. 6, 20913. 10.1038/srep20913 26867973 PMC4751431

